# Monthly Summary

**Published:** 1884-11

**Authors:** 


### Monthly Summary.

---

Anatomical Reasons for Dento-Alveolar Abscess of the Hard Palate.—By dento-alveolar abscess of the hard palate we mean abscess originating in, at or near a tooth, and having as its proximate cause diseased elements in that tooth, and this in contradistinction to alveolar abscess not traceable to nor dependent upon such elements—an affection, by the way, very rarely met with.

As a rule, no matter on which side of the roots of single-rooted teeth, nor which side of the buccal roots of double or triple-rooted teeth an abscess may form, it is more than likely to open over the affected root, and on that side next the lip, or cheek, as the case may be. The same thing is true when an abscess forms on the inermost root of triple-rooted teeth, as the superior molars. The first bony tissue involved is that extremely sponge kind grasped by the roots. This is broken down, the pus burrows into that neighboring tissue offering the least resistance, passes between, and possibly under, the buccal roots, and finally perforates the extremely thin layer of compact bone forming the external plate of the jaw. But an abscess does at times form at or near the palatine root of the superior molars, and opens in the hard palate, and show itself as a fistulous opening in this more plastic but rarely affected appendage. In a case like this an indifferent examination will reveal a squarely vaulted roof, an easily-compressed quantity of connective tissue filling unusually deep depressions in those parts corresponding to the point at which the alveolar process is built upon the jaw proper, and extending from qehind forward the distance comprised between the posterior palatine foramina, which are usually on a line with the farther aspect of the third molar, and the second (sometimes the first) bicuspid. Anatomically these depressions, right and left, are known as the posterior palatine grooves, and are for the lodgment of the palatine glands and transmission of the posterior palatine vessels and descending palatine nerves, all of which lie close to the jaw, and are well covered and protected by the easily-compressed connective tissue just mentioned.

Now where we find such conformation as this, we experience no difficulty in arriving at a conclusion as to why an abscess should open on the palate, hard or soft, rather than on the buccal surface of the maxillary bone—namely, spongy bone in the vicinity of the root, a thin layer of compact tissue at the surface, and this layer, particularly in the young subject› perforated by innumerable minute openings for the transmission of equally minute vessels and nerves. Unquestionably the porosity that characterizes spongy bone, and the many foramna in the compact tissue, though filed with vessels and nerves, offer little resistance to the progress of borrowing pus.

But while abscesses of or in the hard and soft palate may be, and in some few cases are, traceable to the posterior teeth, and because of the condition of things by which they are surrounded, the great majority of abscess of the region in question are traceable to the anterior teeth, and particularly the lateral incisors. Without exception the authorities agree that the lateral incisors are the guilty teeth; but the reasons for this behavior on their part are not only not even hinted at, but in effect confessed as past finding out. Tomes (System of Dental Surgery, '73; p. 486,) says: "For some reason not very apparent, abscess of the hard palate almost invariably proceeds from a lateral incisor." Salter (Dental Pathology and Surgery, '75, p. 240,) says: "Occasionally the matter, in alveolar abscesses, points at the posterior extremity of the hard palate, though the abscess has been occasioned by one of the front teeth, usually the upper lateral incisor. Why this should arise I cannot say: the pathological anatomy of the affection is essentially the same as in ordinary cases, only the canal of the abscess is lengthened out in tne narrow cancellated bone between the two compact plates of the palatal process of the superior maxilla." Other authors are agreed as to the causative tooth, and state the well known facts, but never offer a suggestion as to even a possible reason for their being so.

If we examine a human skull from the under side we will find back of and between the central incisors a depression, slight or great, pierced at the bottom by two, three or (in the symmetrical human skull) four holes. This depression is the anterior palatine fossa, and the holes the foramina which transmit the loft and right naso-palatine nerves and the anterior palatine vessels. When the depth of this fossa is great, and the layer of bone between it and the central incisor thin, there seems to be greater reason for an abscess jointing on the palate than where it usually does—under the lip. And as a further inducement to pus to work that way, we have the inter-maxillary suture, the immediate tissue of which is neither spongy to a great degree, nor very compact—a cross between the two. And this leads to the observation that in the early history of alveolar abscess opening directly over the central incisor, the way from the fistulous opening to the root is not direct, but by way of the suture.

In a further examination of the skull in question "a gelicate linear suture may be seen extending from the anterior palatine fossa to the inlerval between the lateral incisor and the canine tooth. This marks out the inter-maxillary, or incisive bone, which in some animals exists permanently as a separate piece." (Grey, '83, p. 188.) This suture is the dividing line between incisors and other teeth in the lower animals as well as man. A tooth found anterior to this line is in the incisive or premaxillary bone, and by its place of fixation, if for no other reasons, is known to be an incisor. This, to the comparative anatomist, important suture persists in most of the lower animals—Mammals, (sheep, porcupine,) Birds (fowl for example,) Reptiles (crockodile,) Batrachians (frog,) Fishes (perch ;) but in the well-developed adult man it is all but obliterated by the fusing together of the pre- and true-maxillaries. In the infant t is one of the noticeable features of the jaw, and may be raced without much difficulty in some subjects of twenty years or more. But where it persists in the human adult it is confined to the palatal aspect. In fact, we have yet to see a human adult skull that shows this suture from the facial side.

Without further attempt at description of the parts involved, it will be seen that this suture between the pre- and truemaxillary bones furnished the chief anatomical reason for the appeaarnce of dento-alveolar abscess in the palate, when the tooth concerned is a lateral incisor. An abscess forms at the root of a lateral (or it may be a canine, thongh more rarely,) and meeting with less resistance on the side next this suture, breaks down the thin tissue, and the pus burrows, not in the direction of the facial wall, which is perfectly closed by compact tissue, but in the direction of the palate, obstructed in its course only by the thin layer of connective tissue filling the suture. It may continue its course to and through the inner-maxillary suture to the junction of the palate bones, or beyond, and then empty into the mouth or before reaching the inner-maxillary suture it may turn off at a point almost directly back of the root of the canine tooth, follow the posterior palatine groove, and open opposite thd palatine root of one of the molars, thus giving rise to the suspicion that the trouble originated close at hand when in fact it was quite remote.

The conclusions, then, of this effort are, that abscesses of the hard galate are traceable to palatal roots of back teeth, or to the roots of the anterior teeth; that when the back teeth are involved, the roof of the mouth is squarely arched; and as a consequence the quantity of bony tissue between that covering the palatal processes and the root concerned (which is almost invariably the palatal) is extremely thin; that when the front teeth are involved, we may say of the central incisor that its nearness to the inter-maxillary suture, or to the anterior palatine fossa, or both, and the thin and spongy character of the bone separating them, accounts for the position of the abscess; or of the lateral incisor (and more rarely of the canine) that its close proximity to the suture separating the pre-and true-maxillary bones; the thin and spongy or porous character of the intervening bone, the perfectly fused and compact bone on the facial side, and the persistence of the incisive suture on the palatal side, furnish a state of things that accounts for the appearance of dento-alveolar abscess of or in the hard palate, and justifies the further conclusion that hidden things may often be brought to light if we but dig deep enough.—*Odontographic Journal.*

THE TREATMENT OF CARBUNCLE.—Although carbuncle is usually classed among the surgical affections, it comes as properly under the nosological list of the medical practitioner, for it is an affection the treatment of which the general practitioner rarely, if ever, delegates to the specialist in surgery. It appears, however, from a recent clinical lecture delivered by Dr. John Ashhurst, Jr., of Philadelphia, that there exists the danger of incalculable injury from a close conformity to the traditional methods of treatment. A carbuncle is, Dr. Ashhurst states, nothing more than a large boil, differing from a furuncle only in degree. The usual opinion is, we believe, that a carbuncle is an accumulation of boils. This opinion has its origin in the fact of the numerous openings in the carbuncle. These openings are, however, not those of separate boils, Dr. Collins Warren, of Boston, found by a microscopical examination of the skin of the back where carbuncles usually occur, that there are little processes or tubes of fat connecting the deeper tissues with the surface,—columnæ adiposæ. It is along these columns that the pus of the carbuncles, which originate as a phlegmon in the deep cellular tissue, begins to to make its way to the surface, thus causing these separate pimples.

Although carbuncle occurring on the face is usually held to have a fatal termination, such has not been the experience of Dr. Ashhurst. Regardless of the seal of its attack, he maintains that unless occurring in a patient with Bright's disease or diabetes, or in such a situation as to endanger internal organs, death will seldom ensue from carbuncle, except as a result of injudicious treatment. He denounces with considerable vigor the old-fashioned crucial incision. While it has some advantages, these are more than counterbalanced by disadvantages. He speaks also of the cauterization of the carbuncle with a view to causing central sloughing, as of a relic of barbarism, and he regards it as a most fortunate fact that the recommendation of some later surgeons to excise the whole mass of the carbunble has not been received with favor. The method which he now invariably employs is the pressure treatment, after the manner first recommended by Mr. O'Ferrall, an Irish surgeon. He has modified the treatment as originally recommended, by securing the pressure by means of strips of of adhesive plaster applied concentrically as they are used in the treatment of swelled testicle. The whole growth is thus covered, with the exception of the space in the centre to allow the slough to come out. He has found this treatment to yield much better results, both as regards immediate relief of the suffering and the ultimate complete recovery, than any of the other methods which he has seen followed.—*Therapeutic Gazette.*

---

ANÆTHESIA PER RECTUM.—Mr. Daniel Moliere has produced ether narcosis by forcing the ether into the rectum of of the patient. In the first case, that of a young womau of 20, it was administered by means of a Richardson's spray apparatus. The absorption of the ether progressed very slowly, but in the course cf 10 minutes the patient became unconscious. During the operation a few drops ot ether, inhaled from a handkerchief, was sufficient to maintain profound anæthesia. After the operation, which progressed quietly, she vomited the soup that was injected shortly before the operation otherwise there was no disturbance. In the second case the ether was given in a different manner. A rubber tube about the thickness of a finger was introduced into the rectum, and the other end connected with a bottle of ether, which was placed in a vessel of hot water 50°C. The heated ether expanded and forced itself into the rectum, and in the course of 5 minutes the patient was unconscious. The operation to be made was the removal of a tumor from the antrum of Highmore, and in this class of operations about the face, the new procedure is likely to become quite valuable. In the next case the patient had always been a hard drinker, but he became anæsthetized without showing any signs of the stage of excitement.—*Lyon Dedicale.* T.

